# Fifty years of pancreatic islet pathology in human type 1 diabetes: insights gained and progress made

**DOI:** 10.1007/s00125-018-4731-y

**Published:** 2018-09-25

**Authors:** Noel G. Morgan, Sarah J. Richardson

**Affiliations:** Islet Biology Exeter (IBEx), Institute of Biomedical and Clinical Science, University of Exeter Medical School, RILD Building (Level 4), Barrack Road, Exeter, EX2 5DW UK

**Keywords:** Beta cell, Chemokine, Cytokine, HLA class I, Insulin, Insulitis, Islets of Langerhans, Review

## Abstract

Type 1 diabetes is increasing in incidence in many parts of the world and it might be imagined that the pathological processes that underlie disease progression are firmly understood. However, this is not the case; rather, our collective understanding is still surprisingly rudimentary. There are various reasons for this but one of the most important is that the target organ (the pancreas) has been examined at, or soon after, diagnosis in only a small number of cases worldwide over the past half a century. This review provides a summary of some of the insights gained from these studies and highlights areas of ongoing uncertainty. In particular, it considers the process of insulitis (a form of islet inflammation that occurs characteristically in type 1 diabetes) and discusses the factors that may influence the access of immune cells to the beta cells. Attention is also drawn to recent evidence implying that two distinct profiles of insulitis exist, which occur differentially in people who develop type 1 diabetes at increasing ages. Emphasis is also placed on the emerging (and somewhat surprising) consensus that the extent of beta cell loss is variable among people with type 1 diabetes and that many (especially those who are older at onset) retain significant numbers of insulin-producing cells long after diagnosis. We conclude by emphasising the importance of renewed efforts to study the human pancreas at disease onset and consider how the current insights may inform the design of future strategies to slow or halt the rate of beta cell loss.

**Electronic supplementary material:**

The online version of this article (10.1007/s00125-018-4731-y) contains a slideset of the figures for download, which is available to authorised users.

## Historical perspectives

The concept that type 1 diabetes represents a specific disease with a unique aetiology only gained acceptance in the mid-1970s [1] although earlier pioneering studies (mainly by Willy Gepts in the 1960s [2]) had hinted at this conclusion by revealing some of the characteristic pancreatic pathology now known to define it. Progress has remained sluggish, however; largely because a meticulous examination of the target organ is not easily achieved in living individuals. Efforts are underway to improve non-invasive imaging [3] but, in humans, the pancreas remains largely inaccessible and surgical interventions still carry a serious risk. Useful rodent models have been developed to assist in defining the aetiology of type 1 diabetes [4–6] but there are fundamental differences between the rodent and human pancreas with respect to islet architecture [7], innervation [8, 9] and vasculature [8–10], which require caution to be exercised.

**Fig. 1:**
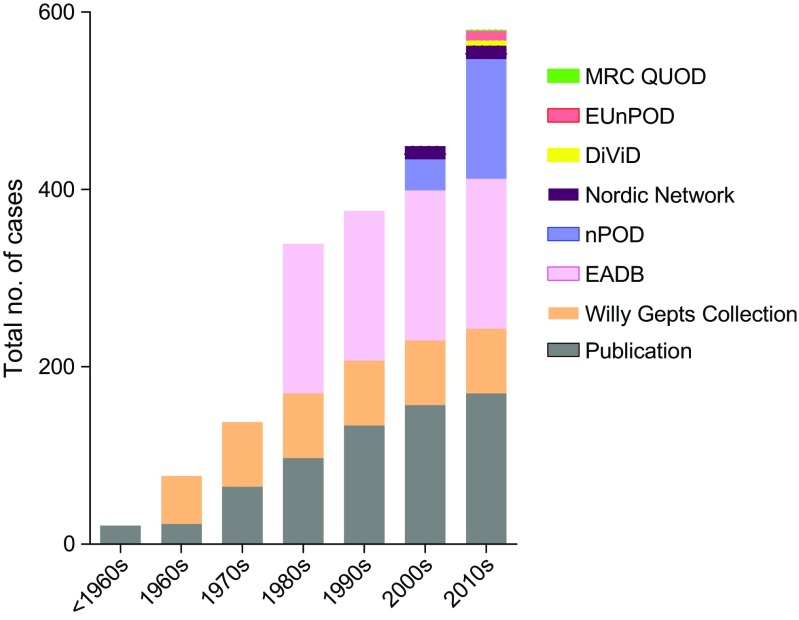
Cumulative number of type 1 diabetes cases that have become available for study over the past 50 years. Fewer than 600 human pancreatic samples are available for study of the aetiopathology of type 1 diabetes in the various collections held across the world. Some form part of larger collections while other samples are cited only in specific publications (defined as ‘Publication’ in the key). These have been accumulated over time and the collection of samples is ongoing in the nPOD, European nPOD (EUnPOD), Nordic Network for Clinical Islet Transplantation and QUOD Biobank initiatives. Despite this, only a small subset of samples come from individuals with recent-onset (≤2 years) disease. DiViD, Diabetes Virus Detection Study. This figure is available as part of a downloadable slideset

**Fig. 2:**
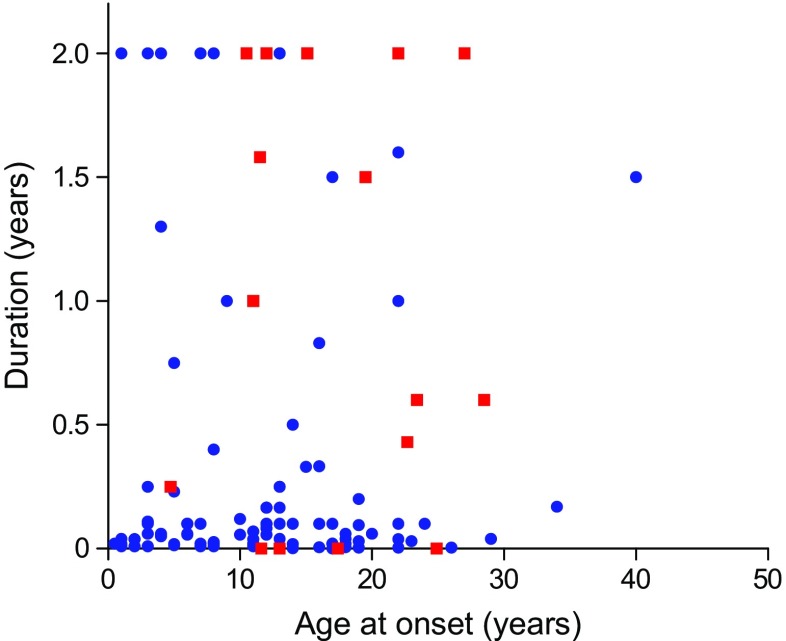
Comparison of pancreas samples available from donors with recent-onset (≤2 years) type 1 diabetes in the two largest collections available for study. Most of the pancreas samples available within the EADB (blue circles) come from individuals who were under the age of 20 years at diagnosis. Many had been diagnosed with type 1 diabetes for less than 6 months at the time of recovery of the gland. By contrast, those in the nPOD collection (red squares) were mainly older at onset. This figure is available as part of a downloadable slideset

Looking back over the last 50 years, it is sobering to realise that fewer than 600 human pancreatic samples in total have been studied from people with type 1 diabetes or are available in pancreatic biobanks (Fig. 1; Table 1). Moreover, only a small subset of these come from young people with recent-onset disease. The largest collections are held in the Exeter Archival Diabetes Biobank (EADB) and the Network for Pancreatic Organ Donors with Diabetes (nPOD) [11]. The EADB is an archival collection comprised of predominantly post-mortem pancreas samples collected from young people (<20 years old) with recent-onset (<2 years) disease who died between 1935 and the mid-1990s [12]. Importantly, it contains the world’s largest collection of pancreas tissue from individuals diagnosed with diabetes under the age of 10 years (a comparison of the two largest collections is shown in Fig. 2; Table 2). However, most specimens were obtained post-mortem and the tissue is of variable quality such that its suitability for certain applications (e.g. RNA sequencing) is limited. By contrast, nPOD tissues are harvested and processed according to optimised standard operating procedures and samples are available for multiple applications [11, 13, 14]. Nevertheless, most nPOD pancreases come from older-onset donors with longer disease duration (Fig. 2; Table 2) and only a single organ is currently available from a donor under the age of 7 years with a short duration (<2 years) of disease. Newer initiatives, including the European INNODIA consortium and a Medical Research Council (MRC)-funded pancreas collection (the Quality in Organ Donation [QUOD] Biobank; coordinated from the University of Newcastle, UK) promise to deliver high-quality samples in the future. Further study of pancreas biobanks remains paramount for an improved understanding of diabetes [15].

**Table 1:** Principal type 1 diabetes pancreas biobanks available for study

Biobank name; location	Number of donors with type 1 diabetes	Examples of other donor types within the collection	Website link
EADB; UK	169	No diabetes AAb^+^ without diabetes, Type 2 diabetes Cystic fibrosis Coxsackie-infected neonates	http://foulis.vub.ac.be/ (accessed 28 August 2018)
nPOD; USA	135	No diabetes AAb^+^ without diabetes Type 2 diabetes Cystic fibrosis related diabetes Pancreatitis Gestational diabetes Pancreatic transplant	www.jdrfnpod.org/ (accessed 28 August 2018)
European nPOD (EUnPOD); Italy	11	No diabetes	NA
Belgium Diabetes Registry, (includes the Willy Gepts Collection); Belgium	73 (datasets for *n* = 22 available online)	No diabetes AAb^+^ without diabetes	www.diabetesbiobank.org/ (accessed 28 August 2018) http://gepts.vub.ac.be/ (accessed 28 August 2018)
Nordic Network for Clinical Islet Transplantation; Sweden	15	No diabetes AAb^+^ without diabetes Type 2 diabetes	http://nordicislets.medscinet.com (accessed 28 August 2018)
MRC QUOD Biobank; UK	1	No diabetes	http://gtr.ukri.org/projects?ref=MR%2FR014132%2F1 (accessed 28 August 2018)

Autoantibody positive, AAb^+^; NA, not available

**Table 2:** Comparison of the type 1 diabetes samples available within the EADB and the nPOD pancreas collection

Variable	EADB	nPOD
Age at onset (years)	11.00 (5.0–16.0)	11.52 (6.2–18.4)
Age at onset (years), range	0.5–40	1–82
Disease duration (years)	0.143 (0.04–3.75)	12.00 (5.5–23.0)
Disease duration (years), range	0–19	0–84
Donors with ≤1 year duration		
*n*	85	11
Age of onset (years)	12.0 (6.0–17.0)	17.4 (11.6–23.0)
Donors ≤7 years old at onset		
*n*	49	48
Duration (years)	0.25 (0.0–6.0)	23.0 (10.5–34.0)
Donors ≤20 years old at onset		
*n*	116	107
Duration (years)	0.17 (0.0–4.0)	14.0 (7.0–24.5)

Data presented as median (interquartile range) unless otherwise stated. Onset and duration data are available for 128 EADB cases and 133 nPOD cases

## Islet structure and composition

**Fig. 3:**
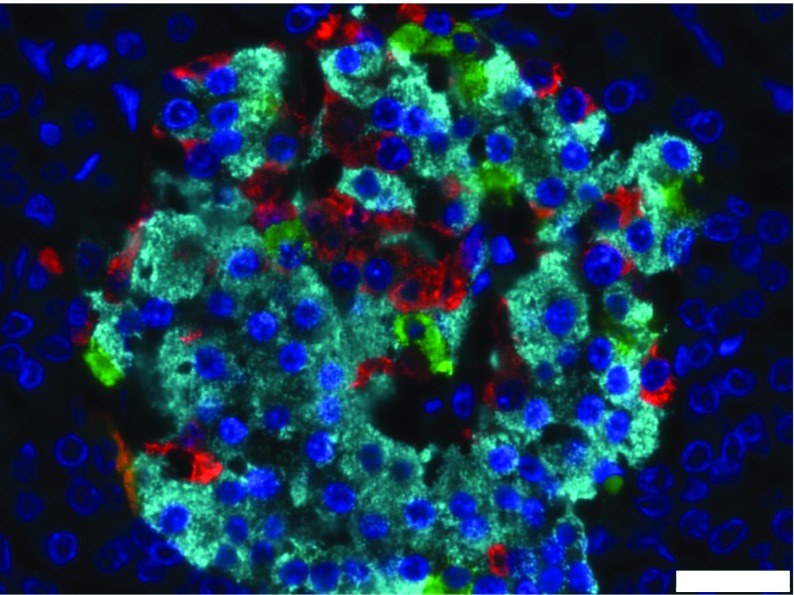
Cellular composition of a human islet of Langerhans from a healthy individual. Individual endocrine cell subtypes were identified by immunofluorescent analysis after staining with antisera directed against insulin (light blue), glucagon (red) and somatostatin (green). Cell nuclei were stained with DAPI (dark blue). Scale bar, 25 μm. The image was captured in our laboratory in Exeter and is from a case held in the EADB collection. This figure is available as part of a downloadable slideset

In humans, islets are widely dispersed across the pancreas, although they may be present at higher density in the head and body of the organ than in the tail [16]. Each islet has a multicellular structure and, in rodent species, the beta cells are localised at the centre with the remaining endocrine cell types arranged in a peripheral ‘mantle’. In human islets, the beta and non-beta endocrine cells occur in a less structured conformation, particularly in larger islets [7, 16] (Fig. 3). Moreover, the proportions of the various endocrine cells are different between species, with a smaller overall percentage of beta cells (50–60%) in humans than in rodents [16, 17].

## Insulitis

**Fig. 4:**
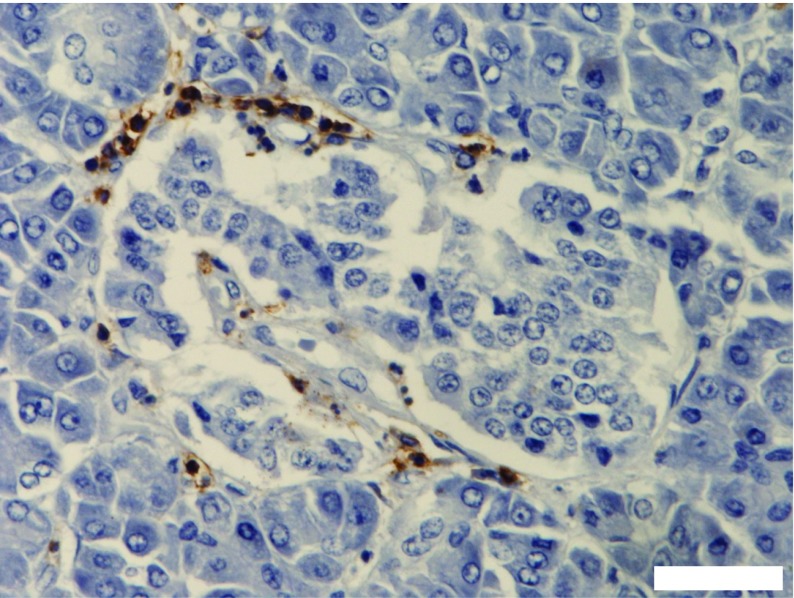
Example of an inflamed islet from a child newly diagnosed with type 1 diabetes. Lymphocytes were immunostained in brown with an antibody directed against CD45. Small numbers of lymphocytes are found within the core of the islet but most are located peripherally, with the majority focused at one pole of the islet. Scale bar, 20 μm. The image was captured in our laboratory in Exeter and is from a case held in the EADB collection. This figure is available as part of a downloadable slideset

An important advance arising from studies of the human pancreas in type 1 diabetes has been the description of insulitis, a process of immune cell infiltration into islets [18] (Fig. 4). This is also seen in animal models but insulitis is found at a lower frequency across the islet population in humans than in rodents, and the proportion of the various individual immune-cell subtypes differ between species (e.g. CD8^+^ T cells dominate human insulitis, whereas CD4^+^ cells are predominant in NOD mice). One note of caution, however, is that some evidence implies that the period spent in intensive care prior to organ recovery can influence the extent of immune-cell infiltration into the human pancreas [18].

Insulitis occurs mainly within the residual insulin-containing islets of people with recent-onset type 1 diabetes and many fewer insulin-deficient islets are inflamed [12, 19–21]. This implies that immune cells are recruited and retained primarily in response to factors emanating from their target beta cells, although the proportion of islets with inflammation varies, not simply in response to beta cell numbers, but also according to the age at disease onset. For example, among individuals diagnosed aged 13 years or older, the proportion of residual insulin-containing islets with insulitis is around 25%, whereas it is much higher (~80%) in those diagnosed in the very early years of life (<7 years of age) [22]. These statistics suggest that young children may have a more aggressive form of the disease. A further implication is that, among older individuals, functional insulin deficiency can occur despite the retention of a significant reserve of the hormone. This points not only to beta cell loss as a cause of type 1 diabetes, but also suggests an insulin-secretory dysfunction among the residual, non-inflamed islets in some individuals. These conclusions are supported by in vitro studies showing that the immediate post-isolation deficit in glucose-induced insulin secretion in the islets of people with type 1 diabetes may improve with time in culture [23].

The occurrence of insulitis is not restricted solely to the period immediately after disease onset but it can also be found in the insulin-containing islets of individuals with long-duration disease [21]. By contrast, it is less clear how long before diagnosis insulitis occurs in people who are progressing to disease. This is much more difficult to evaluate, of course, because prediction of disease onset is still an imperfect art. Nevertheless, it has proved so hard to uncover firm evidence of insulitis in individuals prior to disease onset that In’t Veld has defined the process as an ‘elusive lesion’ [21, 24–26].

### Progression of insulitis

The relative difficulty in identifying insulitis in the human pancreas has led to the publication of an international consensus statement by which insulitis can be defined [27]. This represents a welcome advance since varying definitions have appeared in the literature over time [11, 19, 25, 28–31]. The consensus definition notes that ‘the lesion should be established in a minimum of three islets, with a threshold level of ≥15 CD45^+^ cells/islet before the diagnosis can be made’ [27, 32]. The utility of this statement has been challenged [33], but it remains an internationally accepted working definition [32].

Although the precise composition of the immune infiltrate is variable between islets and among individuals, important categories of insulitis have now been defined. In particular, in humans, CD8^+^ T cells are predominant, whereas CD4^+^ cells are often represented only as a minority population [19, 21, 34, 35]. In addition, the proportion of CD20^+^ cells (B cells) is variable [34–36] since, in very young children (≤7 years of age) these are present in relative abundance in inflamed islets, whereas their proportion is much lower in those who are older at diagnosis [34, 35]. The consequences of this difference (and the factors that underlie it) remain uncertain, but such variability must be considered when designing immunotherapeutic trials intended to reduce the rate of beta cell demise. In addition to lymphocytes, other immune-cell subtypes, including neutrophils [37], mast cells [38] and natural killer cells [39], have been reported to infiltrate the pancreas in, at least, some individuals with type 1 diabetes.

Work in the NOD mouse suggests the existence of sequential ‘stages’ of insulitis [40]; among these is a feature known as ‘peri-insulitis’ in which islets are surrounded by immune cells but not infiltrated by them. This is followed by more complete infiltration, where islets are invaded and the killing of beta cells occurs at high frequency. Equivalent stages are not seen in humans and invasive insulitis is detected only rarely. This suggests either that, in humans, the killing of beta cells is mediated by the very small number of immune (presumably CD8^+^) cells that penetrate into the endocrine cell milieu or, alternatively, that killing does not require direct contact between immune and beta cells. A third possibility also exists, in which the immune cells play (at best) only a minor role in beta cell death. In this context, recent evidence implies that many of the CD8^+^ cells present in insulitic lesions are resident memory cells, which lack a proinflammatory gene expression signature [41]. Nevertheless, it is also clear that some of the influent CD8^+^ T cells are reactive against islet antigens, suggesting an aggressive intent [42–44].

The histological feature referred to above termed ‘peri-insulitis’ mirrors most closely the arrangement of immune cells found in many inflamed islets in human type 1 diabetes. However, unlike the situation in NOD mice, where islets are surrounded by successive banks of lymphocytes, the influent immune cells are fewer in number in human islets and tend to adorn one of the poles [27, 45] (Fig. 4). This distribution suggests that immune cells are initially attracted to the islet environment but, on arrival, they encounter a barrier to entry. This is probably the ‘peri-islet membrane’, a structure that may require considerable re-modelling in order to permit immune cell entry.

## Islet extracellular matrix

The peri-islet membrane forms part of a complex extracellular matrix that serves both to contain the islet cells and to restrict the access of influent immune cells (reviewed in [46]). The structure is sufficiently loose that soluble molecules, including islet hormones, chemokines and cytokines, can penetrate, but it is more restrictive to the passage of cells. It takes the form of a basement membrane consisting of a matrix of collagen (mainly type IV) and a multitude of associated (glyco)proteins, including perlecan and various laminins. The composition of this membrane is very similar in mouse and human islets, although there are important differences in laminin composition [46, 47]. Beneath the basement membrane is an interstitial matrix made up of other collagen isoforms (mostly types I, III and VI) plus fibronectin and large glycoproteins belonging to the fibrillin and matrilin families. Together these represent a formidable barrier to immune cells.

In order to gain entry to the islet milieu, the basement membrane components must be degraded and, in humans, this occurs in a targeted manner close to the point of immune attack, rather than as a more generalised disintegration of the basement membrane around the whole islet [47]. Interestingly, recent modelling of the process using mathematical simulations has suggested that permeation of the islet basement membrane may be the rate-limiting step in beta cell death in type 1 diabetes [48].

Korpos and colleagues have attempted to define the origin and identity of the enzymes responsible for mediating the breakdown of the islet basement membrane during insulitis and, based on both inhibitor and mRNA expression studies using laser capture microdissected islets, they have proposed that cathepsins are prime candidates [47]. High-resolution analysis has led to the conclusion that macrophages and dendritic cells are the likely source of these enzymes and such cells are present in modest numbers during the autoimmune attack. Whether the accompanying lymphocytes play a direct role by also secreting additional proteases, remains to be established.

## Islet chemokine expression

A key question that arises when considering the mechanisms by which immune cells might reach the islet, is the nature of the chemoattractant. It has been widely assumed that islets are induced to secrete chemokines during the initiation phase of beta cell destruction and that relevant immune cells then migrate to the source of these molecules. In support of this, several chemokines have been detected in inflamed islets, including, most frequently (but not exclusively [49]), the (C-X-C motif) ligand (CXCL)10 [50, 51]. This interacts with its cognate receptor, CXCR3, which has been detected on a subset of CD3^+^ cells present in immune infiltrates, including on a proinsulin-directed, autoreactive clone isolated from an individual with type 1 diabetes [50]. It is also known that beta cells can directly produce certain cytokines, including IL-1β [52], which might play a role in mediating their demise.

## Islet HLA class I hyperexpression

A characteristic feature seen in the majority of insulin-containing islets in individuals with type 1 diabetes is hyperexpression of HLA class I [53, 54]. This was first described in early studies by Bottazzo’s group [55] and Foulis et al [53], but has subsequently been confirmed by others [54, 56]. Importantly, HLA class I hyperexpression is not solely restricted to the beta cells in inflamed islets since it occurs in all islet endocrine cells [54], suggesting that the cells are responding to a locally produced agent that is present uniquely in type 1 diabetes. The response is also independent of the simultaneous presence of infiltrating immune cells since it occurs in islets that are inflamed and those that are not [53]. Importantly, hyperexpression of HLA class I does not persist in islets that are deficient in beta cells [45, 53, 54].

Foulis and colleagues were able to correlate HLA class I hyperexpression with the presence of IFN-α in islets studied in situ [57] and subsequent in vitro experiments have confirmed that exposure of isolated human islets to IFN-α causes HLA class I hyperexpression [58, 59]. Set against this, gene expression studies performed on laser captured, microdissected islets from pancreas biopsies recovered from living individuals soon after the diagnosis of type 1 diabetes have suggested that IFN-α levels are not elevated in these samples [51]. Thus, a dilemma remains.

## Beta cell loss in type 1 diabetes

By convention, it has been tacitly assumed that the demise of beta cells consequent to insulitis is mediated by enhanced apoptosis since exposure of human islets (or clonal beta cells) to proinflammatory cytokines, in vitro, leads to apoptosis [60]. Despite this, only modest increases in beta cell apoptosis have been detected in human type 1 diabetes [28] and this may reflect the rapid clearance of apoptotic cells by resident macrophages, which are known to patrol the islet milieu [19]. However, this explanation may also obscure a more fundamental possibility that beta cell apoptosis occurs much less frequently than is supposed. This concept is gaining acceptance in type 2 diabetes, in which beta cell depletion is increasingly attributed to a process of either de- or trans-differentiation, whereby the beta cell phenotype is lost but the cells themselves remain [61]. Levine’s group have provided evidence for the emergence of larger-than-expected numbers of delta cells in human islets during long term type 1 diabetes and have proposed that these may arise by a process of beta cell trans-differentiation [62]. This idea requires further verification but implies that beta cells may not always die in large numbers, after all.

A further feature revealed in recent studies of whole pancreas organs recovered from donors with type 1 diabetes is that the depletion of the islet cells correlates with a more generalised deficit in total pancreatic mass [63]. This was first mooted in the study by Foulis and Stewart [20] and more recent examination of transplant-grade samples from within the nPOD collection has revealed an ~40% reduction in relative pancreas weight in type 1 diabetes vs age matched controls [64]. Thus, the traditional view that the pathological effects associated with type 1 diabetes are restricted solely to the endocrine compartment of the tissue may require revision and increasing evidence suggests that imbalances in immune-cell infiltration also extend more widely across the gland [37, 64].

## Conclusion

Our understanding of the aetiopathology of type 1 diabetes has advanced greatly since the pioneering studies by Gepts [2], mainly thanks to the opportunity to study additional rare and precious pancreas samples recovered from individuals with the disease. Nevertheless, the task is not complete and the more we look, the more the accepted wisdom is challenged. It is critical, therefore, that such work continues since only then will new opportunities for therapy and disease prevention emerge.

## Electronic supplementary material

ESM Slideset of figures (PPTX 1.84 mb)

## Data Availability

The datasets generated and/or analysed during the current study are available from the corresponding author on reasonable request.

## Abbreviations

CXCL: (C-X-C motif) ligand
EADB: Exeter Archival Diabetes Biobank
MRC: Medical Research Council
nPOD: Network for Pancreatic Organ Donors with Diabetes
QUOD: Quality in Organ Donation
