# Supplementary material for: Fifty years of pancreatic islet pathology in human type 1 diabetes: insights gained and progress made
Source: Diabetologia. 2018 Sep 25;61(12):2499–506. doi: 10.1007/s00125-018-4731-y (PMC6223849; doi:10.1007/s00125-018-4731-y)
Supplement: Supplementary file 1 — (PPTX 1.84 mb) [file 125_2018_4731_MOESM1_ESM.pptx]

## Slide 1
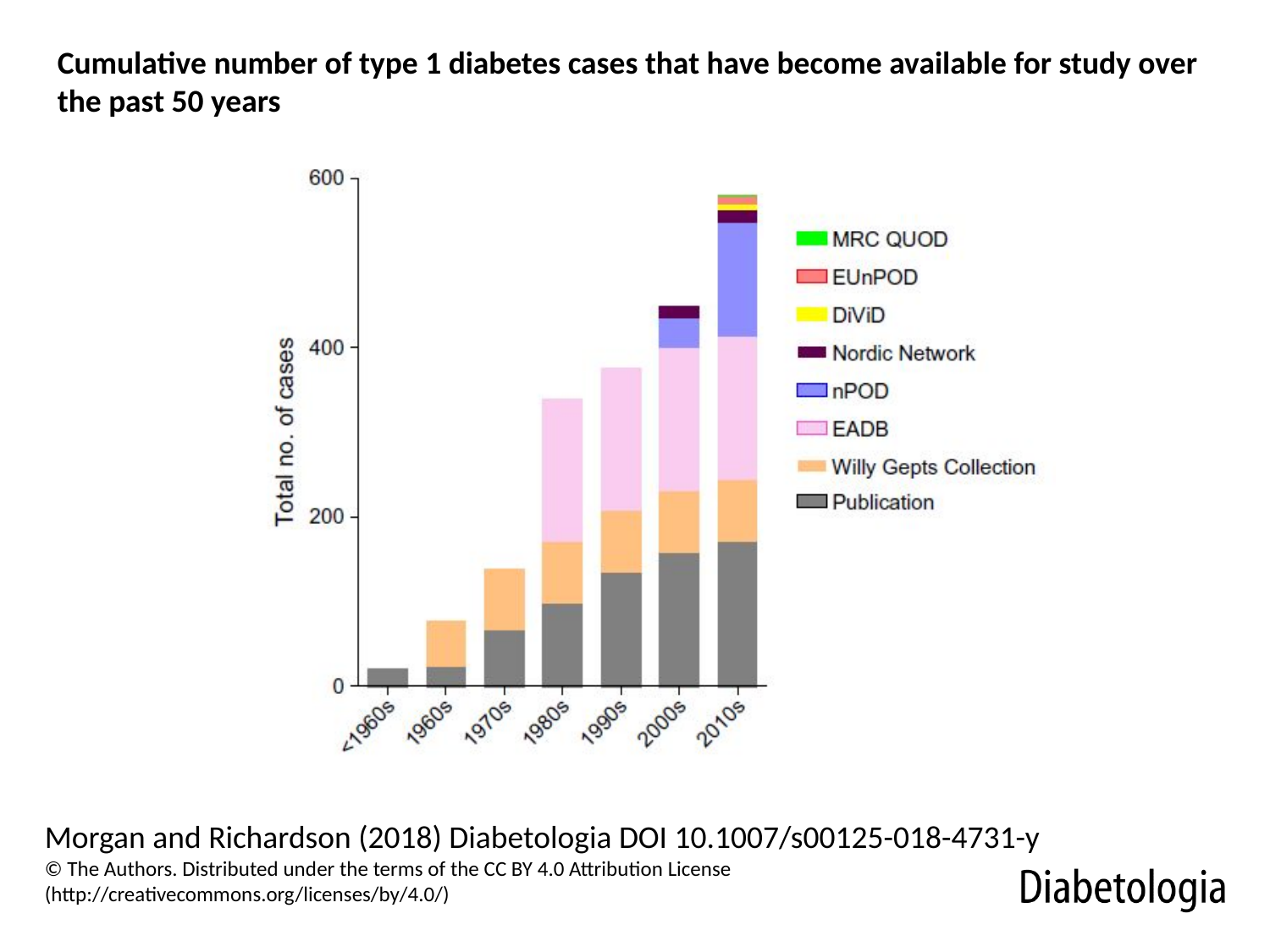

Cumulative number of type 1 diabetes cases that have become available for study over the past 50 years
Morgan and Richardson (2018) Diabetologia DOI 10.1007/s00125-018-4731-y
© The Authors. Distributed under the terms of the CC BY 4.0 Attribution License (http://creativecommons.org/licenses/by/4.0/)

## Slide 2
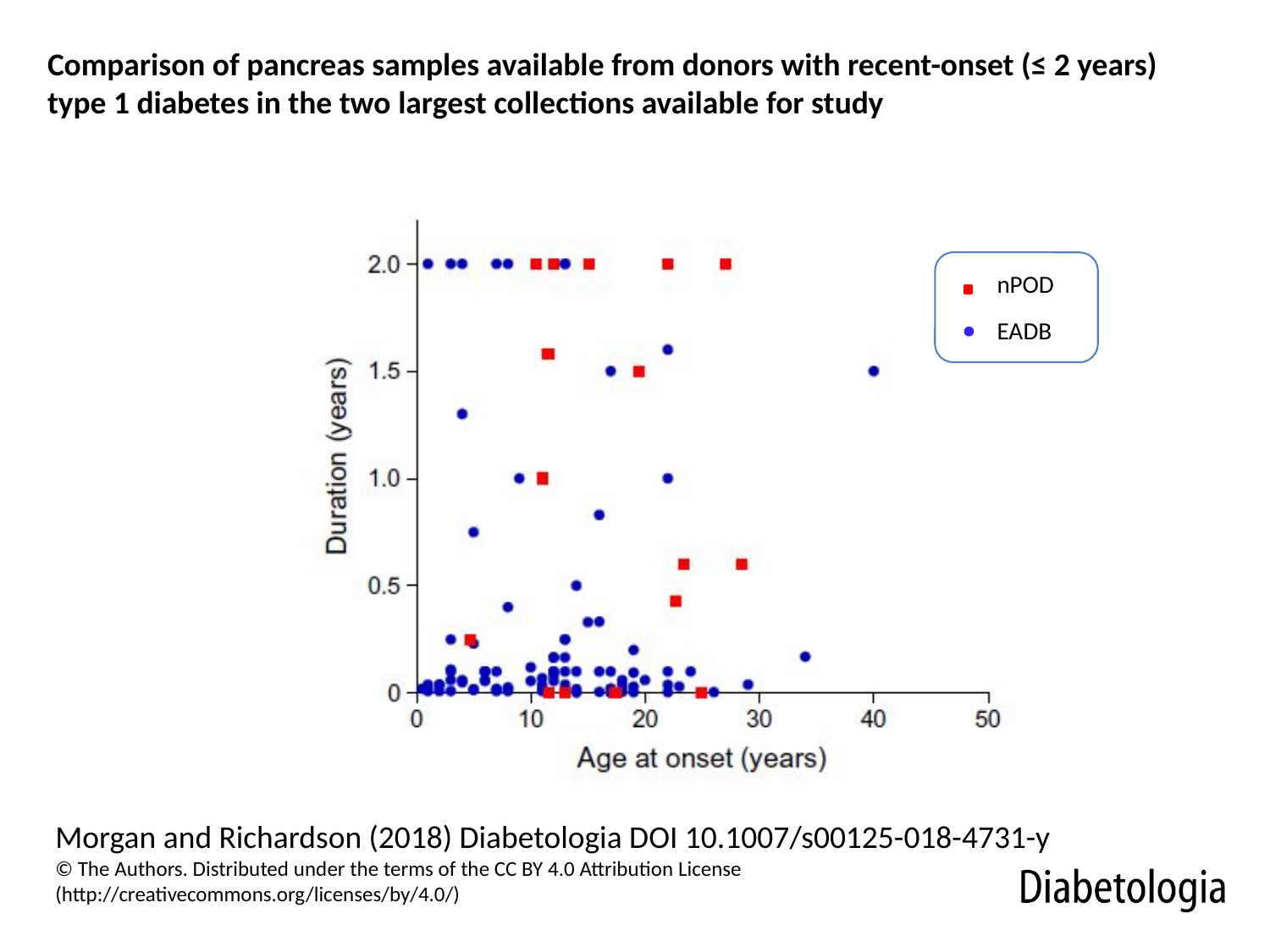

Comparison of pancreas samples available from donors with recent-onset (≤ 2 years)
type 1 diabetes in the two largest collections available for study
nPOD
EADB
Morgan and Richardson (2018) Diabetologia DOI 10.1007/s00125-018-4731-y
© The Authors. Distributed under the terms of the CC BY 4.0 Attribution License (http://creativecommons.org/licenses/by/4.0/)

## Slide 3
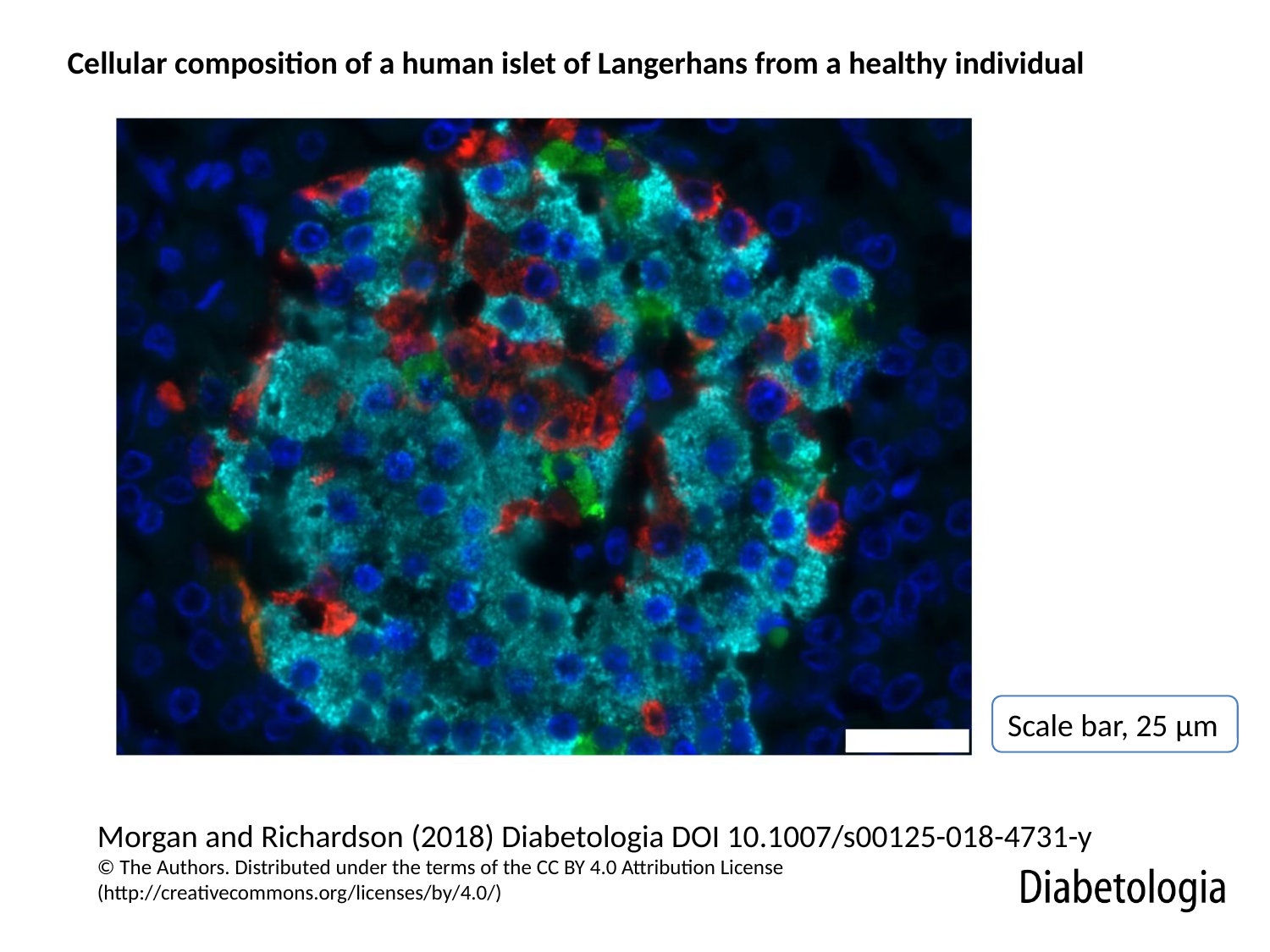

Cellular composition of a human islet of Langerhans from a healthy individual
Scale bar, 25 μm
Morgan and Richardson (2018) Diabetologia DOI 10.1007/s00125-018-4731-y
© The Authors. Distributed under the terms of the CC BY 4.0 Attribution License (http://creativecommons.org/licenses/by/4.0/)

## Slide 4
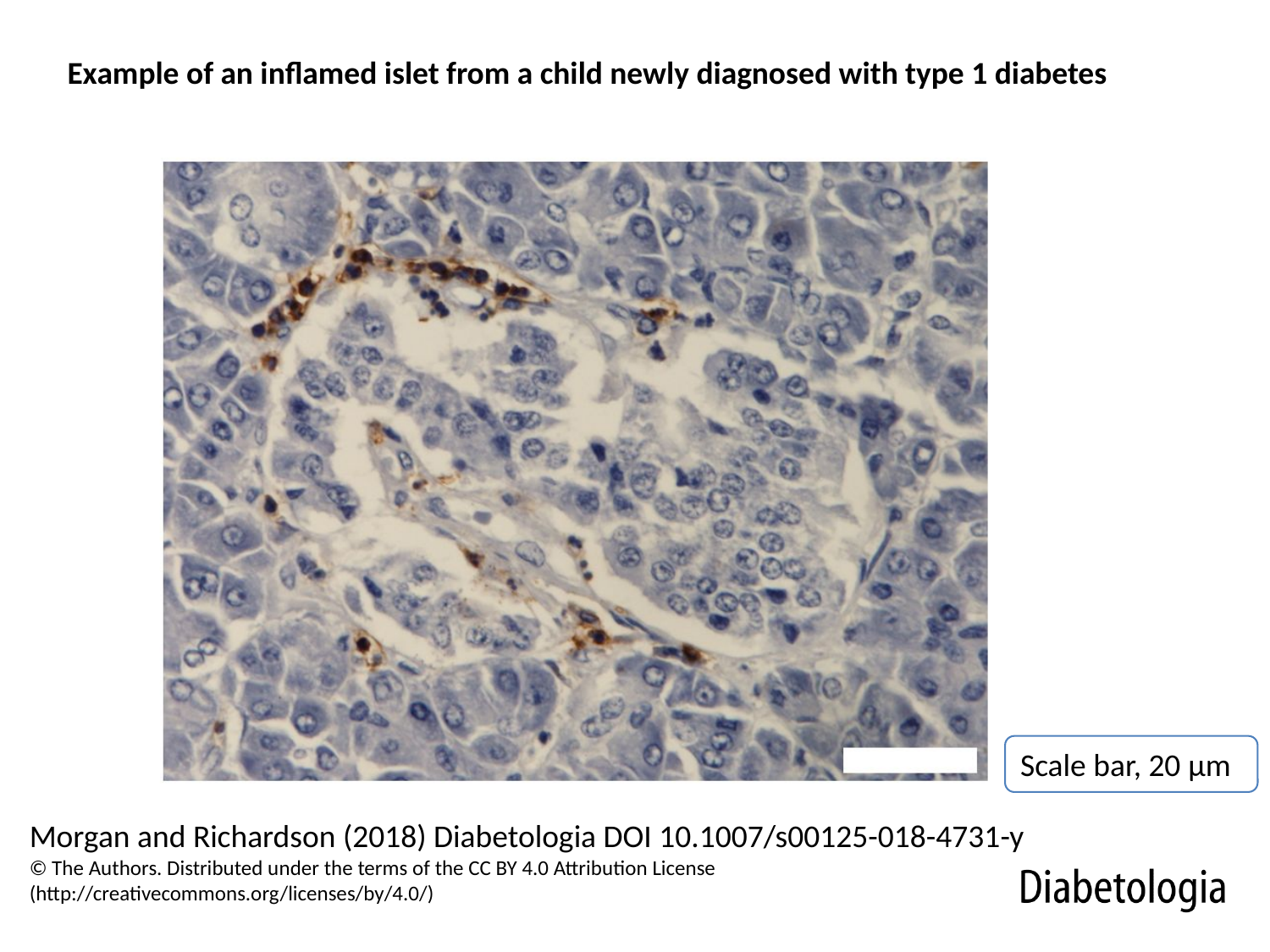

Example of an inflamed islet from a child newly diagnosed with type 1 diabetes
Scale bar, 20 μm
Morgan and Richardson (2018) Diabetologia DOI 10.1007/s00125-018-4731-y
© The Authors. Distributed under the terms of the CC BY 4.0 Attribution License (http://creativecommons.org/licenses/by/4.0/)
